# The association between oral hygiene and metabolic dysfunction-associated steatotic liver disease – a systematic review

**DOI:** 10.1186/s12903-026-09067-y

**Published:** 2026-07-08

**Authors:** Antonia Gerold, Felicitas-Sophie Walter, Jacqueline Waller, Boris Ferger

**Affiliations:** Institute of Experimental and Clinical Pharmacology, Toxicology and Pharmacology of Natural Products, Ulm University Hospital, Ulm, Germany

**Keywords:** MASLD, NAFLD, Fatty liver, Oral hygiene, Tooth brushing, Systematic review

## Abstract

**Background:**

Given the increasingly evident links between oral health and systemic health, the aim of this review was to investigate whether oral hygiene practices influence the risk or progression of metabolic dysfunction-associated steatotic liver disease (MASLD). The findings support the potential importance of good oral hygiene and regular dental visits, especially for patients with MASLD.

**Methods:**

This systematic review was registered with PROSPERO (CRD420251041530), and PubMed, Scopus, Embase and Web of Science were used for literature research up to June 2026. In accordance with the Preferred Reporting Items for Systematic Reviews and Meta-Analyses (PRISMA) guidelines, screening, study selection and quality check were performed using Rayyan and the Joanna Briggs Institute (JBI) critical appraisal checklists. Studies assessing the potential link between the frequency of oral hygiene procedures and the incidence, prevalence or progression of MASLD were selected for a systematic synthesis. The old terms non-alcoholic fatty liver disease (NAFLD) and non-alcoholic steatohepatitis (NASH) were considered as well.

**Results:**

A total of 570 results were identified of which six studies met the inclusion criteria. Five of them reported a correlation between higher tooth brushing frequency and a lower incidence, prevalence or progression of NAFLD/NASH compared to individuals with lower tooth brushing frequency. Notably, one study revealed significantly worse liver parameters among NASH patients without regular dental visits than among those who visited a dentist more than once a year.

**Conclusions:**

An association between oral hygiene habits and NAFLD/NASH was found in all included studies. A greater frequency of daily tooth brushing as well as regular dental visits might have beneficial effects on both the development and progression of fatty liver disease. However, there is not enough evidence to establish causality yet and the impact of confounding factors must be further investigated.

**Clinical trial registration:**

Not applicable

**Supplementary Information:**

The online version contains supplementary material available at 10.1186/s12903-026-09067-y.

## Background

Growing evidence shows that metabolic dysfunction-associated steatotic liver disease (MASLD) affects not only hepatic tissue but has an association with oral health conditions such as periodontitis and tooth loss [1]. With a global prevalence of approximately 30% and increasing tendency, MASLD, also known as non-alcoholic fatty liver disease (NAFLD), has become the leading reason for chronic liver disease nowadays [2–4]. Overnutrition and a lack of physical exercise, especially in the context of the so-called “western diet”, are identified as risk factors for fatty liver disease [5]. Its progressive nature and close relation with other liver-related and extrahepatic complications such as cirrhosis, hepatocellular carcinoma, obesity, diabetes and cardiovascular diseases, as well as its rising global financial load make it a condition of increasing interest [6]. Also in dental medicine, MASLD has received heightened attention due to its connection to oral conditions such as periodontitis, as discussed by several systematic reviews and meta-analyses [1, 7, 8]. While the association between periodontitis and MASLD has been extensively studied, the potential relationship between MASLD and oral hygiene, as an important modifiable factor for oral health, remains unclear.

MASLD, alongside alcohol-associated liver disease (ALD) and others, belongs to the group of steatotic liver diseases (SLD) [9]. Both forms are characterised by an excessive accumulation of triglycerides in the liver [9]. MASLD comprises simple hepatic steatosis as well as the more progressive form, metabolic dysfunction-associated steatohepatitis (MASH), which is characterised by lobular inflammation with histological “ballooning” of hepatocytes and can be concomitant with fibrosis up to cirrhosis [9].

Owing to the perceived stigmatisation by the terms “fatty” and “non-alcoholic” the old term “non-alcoholic fatty liver disease” was recently replaced by “metabolic dysfunction-associated steatotic liver disease” [10]. According to the new definition, the disease profile of MASLD corresponds to that of NAFLD, but additionally requires the presence of at least one out of five cardiometabolic risk factors [10]. Thus, MASH was introduced as the new term for non-alcoholic steatohepatitis (NASH) [10]. As it can be assumed that the new term mostly comprises the entire cohort of patients with NAFLD, the terms NAFLD and MASLD, as well as NASH and MASH, are used synonymously in the following [11].

The aetiology of MASLD is complex and multifactorial. It is commonly accepted that there is a close association with the metabolic syndrome [12], obesity [13] and diabetes [14–16]. Hepatic steatosis is ultimately the result of impaired hepatic lipid metabolism for various possible reasons [17]. Excessive accumulation of fatty acids and triglycerides in liver cells causes hepatic steatosis [18]. When inflammatory and fibrogenetic processes occur, the condition may progress and result in fibrosis and/or steatohepatitis [19].

Recent studies have identified associations between fatty liver disease and various oral health conditions, including periodontitis [20], tooth loss [21], and alterations in the oral microbiome, such as elevated levels of the periodontal pathogen Porphyromonas gingivalis [22]. Rising evidence suggests that metabolic syndrome, type 2 diabetes mellitus and MASLD are interconnected through systemic inflammation linking it to periodontal disease with oral and gut dysbiosis [23]. As oral hygiene measures play a crucial role in maintaining oral health and preventing periodontal disease, they may also be associated with systemic inflammatory conditions such as MASLD. Against this background, the question arises whether oral health, and therefore oral hygiene measures such as tooth brushing, may influence liver health. Although some studies have investigated this connection, the available evidence has not been systematically synthesised yet. This systematic review therefore aims to examine whether the frequency of daily tooth brushing and the number of annual dental check-ups are related to the development and progression of MASLD.

## Materials and methods

The whole review process followed the Preferred Reporting Items for Systematic Reviews and Meta-Analyses (PRISMA) guidelines to ensure a transparent and methodologically sound approach [24]. The review was further registered with the International Prospective Register of Systematic Reviews PROSPERO on 30th April 2025. The registration was confirmed on 9th May 2025, under the number CRD420251041530. Additionally, a review protocol was designed in accordance with the PRISMA-P elaboration and explanation paper, that will be available on PROSPERO after publication [25]. The full search strategy, search term and additional information on data extraction are provided in Supplemental File 1.

### Eligibility criteria

The aim of this review was to investigate whether oral hygiene practices affect the risk of MASLD in healthy individuals as well as the course of disease in patients with MASLD. To develop a research question and search strategy the PICO (“participants, interventions, comparison, outcome”) scheme was used.

Included participants were individuals suffering from MASLD including simple steatosis and steatohepatitis with or without fibrosis or cirrhosis. Non-exposed individuals were likewise enrolled, provided they did not present with chronic liver diseases of aetiologies other than MASLD. There were no restrictions on age, sex or ethnicity in any of the groups. Subjects with other causes of chronic liver disease apart from MASLD were excluded.

Interventions of interest included the frequency of oral health care measures such as daily tooth brushing and annual dental visits. Other practices of oral hygiene were also considered if present. Individuals with a lower frequency of daily tooth brushing, annual dental visits or any further oral hygiene practice were compared to those with a higher frequency, respectively. Any kind of non-surgical or surgical periodontal treatment was not considered as it was out of scope of this review.

The evaluated outcome was the incidence, prevalence or progression of MASLD in individuals with higher versus lower frequencies of oral hygiene practices.

Both randomised and non-randomised study types such as randomised controlled trials, cohort, case-control, cross-sectional and longitudinal studies as well as interventional studies assessing the relation between oral hygiene procedures and the incidence and progression of MASLD were included. Studies using the old terms NAFLD/NASH were also included. Systematic or different forms of reviews and meta-analyses as well as non-human studies were not included.

### Search strategy

The initial search was carried out on PubMed (MEDLINE database) in November 2025, using the term “((oral hygiene) OR (tooth brushing) OR (dental visit) OR (periodontal)) AND ((fatty liver) OR (mash) OR (nash))”. Literature search was extended to multiple databases in June 2026, including Scopus, Embase (access via Ovid) and Web of Science, to minimise the risk of missing relevant studies and to increase the validity of this review. The terms “oral hygiene”, “tooth brushing”, “dental visit” and “periodontal” were intended to cover all areas related to oral hygiene. “Fatty liver”, “mash” and “nash” aimed to encompass the full scope of MASLD. The search term was slightly modified for each database as shown in Supplemental File 1. Only studies in English and German language were selected and animal studies, reviews or meta-analyses were excluded. There was no restriction on the publication period.

### Screening and study selection

Study selection was conducted by two authors independently screening the titles and abstracts of all studies based on the previously described selection criteria. The full texts of the preselected trials were further examined for eligibility. The two authors were blinded to each other’s decision and any disagreement was resolved by a third team member. The decisions were recorded via the platform Rayyan. All decisions were made manually by the author team without the use of automation tools.

### Data extraction and synthesis

Data extraction from the full texts was performed by AG using a standardised form. A second team member, FW, independently extracted the data from a subset of randomly selected studies to check on reliability and consistency. Disagreements were resolved by a third team member, JW. The extracted data for each study were organised and a narrative synthesis of the findings was tabulated. Due to the heterogeneity of the included studies, no meta-analysis was conducted. There were no subgroup analyses.

### Study quality

The risk of bias assessment of the included studies was carried out by AG. FW independently checked the decisions. For this purpose, the Joanna Briggs Institute (JBI) critical appraisal tool for cross-sectional and cohort studies was used [26]. The Newcastle-Ottawa scale for cohort and case-control studies as mentioned in the review protocol was not applied, as there were only one cohort and five cross-sectional studies, but no case-control studies included. The study quality was measured according to the number of satisfied questions (questions answered by “yes”) and was rated as high (> 80%: 7–8/8 or 9–11/11 questions answered by “yes”), moderate (> 60%: 5–6/8 or 7–8/11) or acceptable (≤ 60%: 0–4/8 or 0–6/11).

As no meta-analysis was performed, the strength and limitations of the included studies were discussed descriptively to evaluate their certainty of evidence.

## Results

**Fig. 1:**
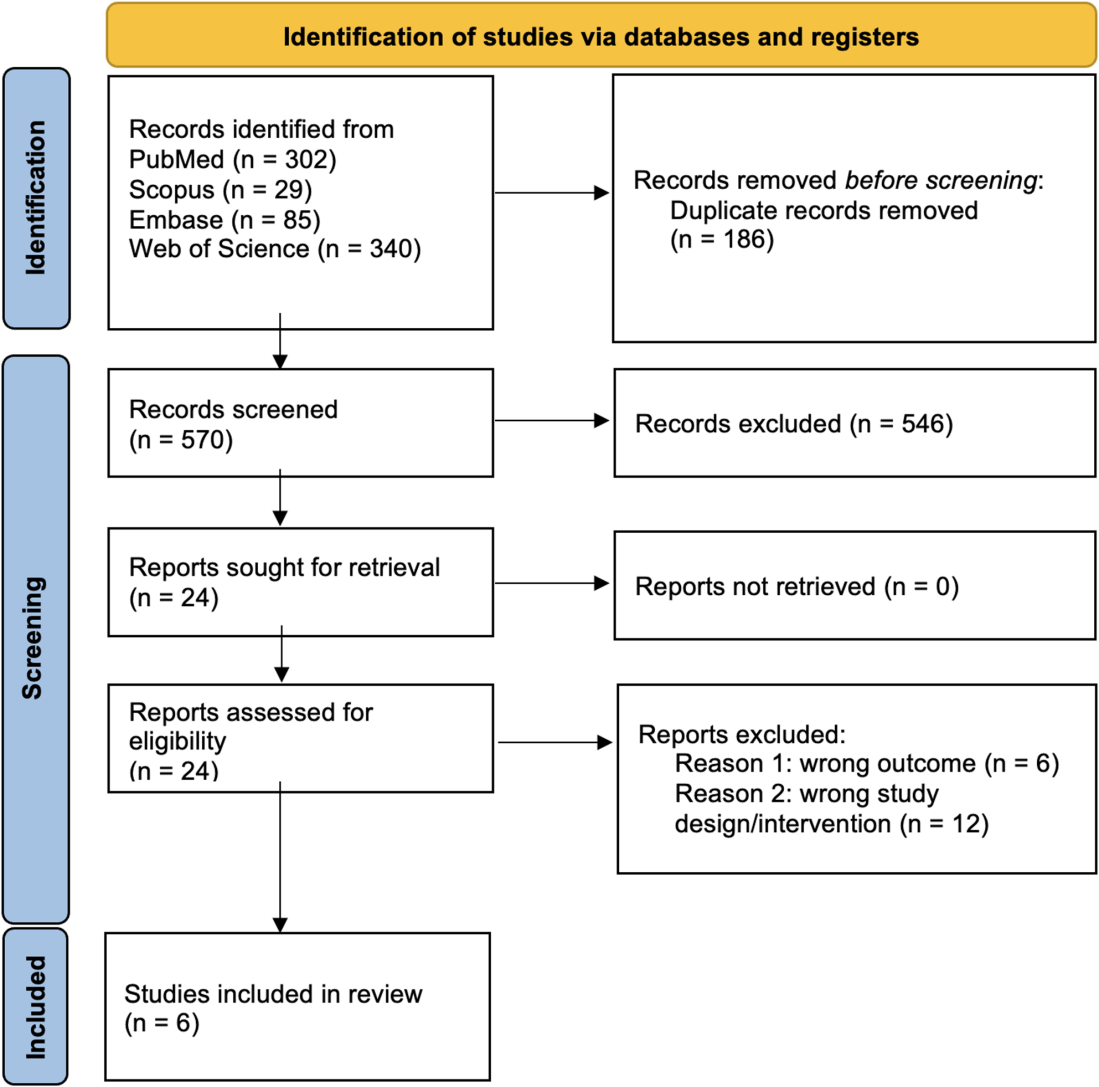
PRISMA flow diagram showing the study selection process

The search delivered a total of 570 results after the removal of duplicates as shown in Fig. 1. Six studies examining the associations between oral hygiene measures and NAFLD/NASH met the eligibility criteria. Those six studies comprised one cohort, four cross-sectional studies and one trial with a hybrid cross-sectional/cohort design from Korea, Japan, Israel, Turkey, Germany and Iran.

### Study quality

The risk of bias assessment is presented in Tables 1 and 2. All included studies were of moderate or high quality. Among the five cross-sectional studies, two met seven of the eight JBI criteria and were rated as high, whereas two met six out of eight, and one met five out of eight, which indicates moderate quality. The cohort study fulfilled seven out of the eleven criteria and therefore was of moderate quality. Details on the risk of bias assessment can be found in Supplemental File 2.

**Table 1:** Quality assessment based on the JBI critical appraisal checklist for analytical cross-sectional studies

First author, year of publication	Q1	Q2	Q3	Q4	Q5	Q6	Q7	Q8	Total score
Kim, 2021 [30]	1	1	0	1	1	1	1	1	7/8
Ram, 2022 [32]	1	1	0	unclear	1	1	unclear	1	5/8
Keklikkiran, 2023 [29]	1	1	0	1	1	1	1	1	7/8
Pischke, 2023 [27]	1	1	0	1	1	0	1	1	6/8
Gheisary, 2026 [28]	1	1	0	1	1	unclear	1	1	6/8

Questions 1–8 (Q1-8): Answers: “yes” = 1, “no” = 0, unclear, not applicable

Total score of ≥ 7/8 indicates high, 5–6/8 moderate, and ≤ 4/8 low quality

**Table 2:** Quality assessment based on the JBI critical appraisal checklist for cohort studies

First author, year of publication	Q1	Q2	Q3	Q4	Q5	Q6	Q7	Q8	Q9	Q10	Q11	Total score
Yamamoto, 2021 [31]	1	1	0	1	1	1	1	not applicable	unclear	unclear	1	7/11

Questions 1–11 (Q1-11): Answers: “yes” = 1, “no” = 0, unclear, not applicable

Total score of ≥ 9/11 indicates high, 7–8/11 moderate, and ≤ 6/11 low quality

### Sample number and participant characteristics

The number of participants differed considerably among the six studies and ranged from 32 [27], to 86 [28], 1,156 [29], 4,259 [30], 25,804 [31], and up to 132,529 [32], which means a total of 163,866 participants. Only adult participants were included in the studies. Detailed study and participant characteristics can be found in Table 3.

**Table 3:** Characteristics of the studies included in the review

First author, year	Country	Aim	Study design	Sample number	Mean age and Gender	Inclusion criteria
Kim, 2021 [30]	Korea	evaluation of the association between daily oral hygiene measures and NAFLD prevalence	retrospective, cross-sectional	4,259	not stated	> 18 years; exclusion of individuals with cancer, liver or kidney diseases, pregnancy, or high alcohol consumption (> 30 g/day (d))
Yamamoto, 2021 [31]	Japan	evaluation of the association between daily tooth brushing (tb) frequency and NAFLD incidence among healthy individuals	retrospective, longitudinal cohort	25,804	45.2 years; 26.7% male	individuals without baseline NAFLD (at initial health check-up), with at least 2 health check-ups during follow-up, no alcohol consumption
Ram, 2022 [32]	Israel	examination of the association between NAFLD prevalence and dental parameters (e.g. tb frequency)	retrospective, cross-sectional	132,529	not stated	individuals with and without NAFLD; > 18 years; Israeli military staff
Pischke, 2023 [27]	Germany	investigation of the association between liver parameters and frequency of dental check-ups among individuals with NASH	hybrid design: prospective cohort with cross-sectional analysis	32	53.0 ± 13.2 years; 50% male	diagnosed adult NASH patients
Keklikkiran, 2023 [29]	Turkey	investigation of the association between tb frequency and risk of cirrhosis development among patients with NAFLD	retrospective, cross-sectional	1,156	48.0 ± 11.1 years; 51% male	adults with NAFLD diagnosis, exclusion of individuals with other cause of chronic liver disease
GHEISARY, 2026 [28]	Iran	comparison of oral hygiene habits of patients with NAFLD and healthy individuals	comparative cross-sectional	86 (NAFLD group: *n* = 43, control group: *n* = 43)	30.4 ± 3.6 years; 59.3% male	NAFLD group: adults with NAFLD, exclusion of individuals with high alcohol consumption, other liver diseases or cardiometabolic complications/disorders (e.g. diabetes), major weight loss or bariatric surgery in the past control group: adults without liver steatosis

### Exposure

As shown in Table 4, four studies assessed the influence of daily tooth brushing frequency [28, 29, 31, 32], one examined the frequency of annual dental appointments [27], and one investigated both [30], all relying on self-reported data from participants. Gheisary et al. and Kim et al. additionally evaluated the use of further oral hygiene products, as Kim et al. did not assess their frequency this was not considered in the review [28, 30].

**Table 4:** Exposure, outcome and diagnostic tools used in the included studies

First author, year	Exposure (oral hygiene measure)			Evaluated outcome			Diagnostic tools
	tooth brushing	other	dental visits	prevalence	incidence	progression	
Kim, 2021 [30]	≤ 1x/d, 2x/d, ≥ 3x/d	-	at least once in the last year (y)): yes/no	NAFLD	-	-	FLI ≥ 60 defined as NAFLD
Yamamoto, 2021 [31]	≤ 1x/d, 1-2x/d, 3x/d	-	-	-	NAFLD	-	ultrasonography
Ram, 2022 [32]	≥ 1/d: yes/no	-	-	NAFLD	-	-	not stated
Pischke, 2023 [27]	-	-	< 1x/y, 1x/y, > 1x/y	-	-	NASH	liver stiffness values (fibroscan), AST, and MELD score
Keklikkiran, 2023 [29]	< 1x/d, 1x/d, 2x/d, after every meal	-	-	-	-	NAFLD (defined as cirrhosis prevalence)	LSM (transient elastography): LSM ≥ 12 kPa defined as cirrhosis
GHEISARY, 2026 [28]	never, < 1x/d, 1x/d, 2x/d, > 2x/d	flossing: never, < 1x/d, 1x/d, 2x/d, > 2x/d mouthwash: never, 1x/month, 1x/week, 1x/d	-	NAFLD	-	-	sonography

### Outcome

The evaluated outcomes were the prevalence of NAFLD, the incidence of NAFLD as well as the course of disease among patients already suffering from NAFLD/NASH (Table 4). The presence of NAFLD was either measured by Fatty Liver Index (FLI) [30], liver stiffness measurement (LSM) [27, 29] or ultrasound [28, 31]. Pischke et al. additionally assessed other liver parameters, such as aspartate aminotransferase (AST) levels and the Model for End-stage Liver Disease (MELD) score [27].

### Findings

In all five studies that assessed tooth brushing frequency, there was a significant correlation between higher frequency of daily tooth brushing and a lower risk, prevalence or progression of NAFLD/NASH (Table 5). The study that assessed only the number of annual dental visits revealed significantly increased liver parameters among NASH patients without regular dental visits [27].

**Table 5:** Main findings of included studies

First author, year	Main findings
Kim, 2021 [30]	• association of decreasing FLI values with increasing tb frequency (*p* < 0.001): • significant negative correlation of high FLI values (≥ 60) with higher tb frequency compared to tb ≤ 1x/d in adjusted models (*p* = 0.02) • no significant correlation when additionally adjusting for diabetes mellitus or regular dental check-ups (*p* > 0.05) • no significant correlation between regular dental check-ups and OR for FLI ≥ 60 compared to no regular dental check-ups (*p* > 0.05)
Yamamoto, 2021 [31]	NAFLD risk decreases in adjusted models with increasing tb frequency compared to tb ≤ 1x/d (*p* < 0.05)
Ram, 2022 [32]	• significant relation of higher tb frequency (≥ 1x/d) with lower NAFLD prevalence (*p* < 0.001) • loss of statistical significance between NAFLD and tb frequency (*p* > 0.05) when controlling for socio-demographic parameters, health-related risk habits (e.g. smoking), conditions associated with the metabolic syndrome, and auxiliary (blood) test results
Pischke, 2023 [27]	significantly increased liver parameters (AST, MELD score and liver stiffness values) among NASH patients without regular dental visits (≤ 1x/y) compared to those with regular check-ups (> 1x/y)
Keklikkiran, 2023 [29]	association of lower tb frequency (≤ 1/d) with higher risk for LSM ≥ 12 kPa compared to tb 2x/d or after every meal (*p* = 0.0236)
GHEISARY, 2026 [28]	lower frequencies of tb, flossing and using mouthwash in the NAFLD group compared with the control group

Kim et al. demonstrated that participants with higher tooth brushing frequency had lower FLI scores [30]. In adjusted models controlling for covariates the prevalence of NAFLD, defined as FLI scores of 60 or more, was significantly decreased among those who brushed their teeth twice a day or more compared to those with a tooth brushing frequency of once a day or less. This association lost its statistical significance when additionally controlling for the presence of diabetes mellitus, though. There was also no significant correlation between the frequency of dental check-ups and the prevalence of NAFLD.

Yamamoto et al. investigated the risk of NAFLD among healthy individuals at baseline in relation to their daily tooth brushing frequency during follow-up [31]. The incidence of NAFLD significantly decreased with increasing tooth brushing frequency. Participants who brushed their teeth more than once a day presented a reduced risk of developing NAFLD.

Ram et al. reported a negative association between increased tooth brushing frequency and NAFLD prevalence, too [32]. However, this correlation lost its statistical significance when covariates such as socio-demographic factors, health related risk habits, conditions related to the metabolic syndrome, and the results of auxiliary tests were controlled for.

Pischke et al. compared liver parameters among a cohort of diagnosed NASH patients [27]. Those without regular dental visits had significantly increased values of liver parameters such as AST, MELD and LSM than those with dental check-ups more often than once a year.

Keklikkiran et al. examined the relation between tooth brushing frequency and disease progression, defined as the incidence of cirrhosis, among patients with baseline NAFLD [29]. Compared with tooth brushing twice a day or more, a tooth brushing frequency of once a day or less was significantly associated with a greater risk of liver cirrhosis.

Gheisary et al. compared oral hygiene habits between individuals with NAFLD and healthy controls [28]. The NAFLD group showed lower frequencies of daily tooth brushing, flossing or using mouthwash compared with the control group. While 18.6% of the NAFLD group never brushed their teeth, 58.1% of them never used dental floss, and 72.1% never used mouthwash, all controls brushed their teeth once a day or more and used floss or mouthwash at least occasionally.

## Discussion

All analysed studies demonstrated an association between oral hygiene habits and NAFLD. Frequent tooth brushing as well as regular dental visits seemed to have beneficial effects on both the development and progression of fatty liver disease. These findings suggest that oral health plays a significant role in overall health, including liver health. Five of the included studies reported a correlation between higher tooth brushing frequency and lower risk, prevalence or progression of NAFLD [28–32]. Statistical significance was lost in two cases, though, when additionally controlling for diabetes mellitus [30] or socio-demographic parameters and other health-related conditions or habits [32]. Pischke et al. demonstrated a deterioration in liver parameters, signifying a worse liver status, among NASH patients who did not attend the dentist on a regular basis [27], whereas Kim et al. did not identify a significant correlation between regular dental check-ups and NAFLD prevalence [30].

In the past few years, growing evidence has suggested a possible link between the incidence and progression of MASLD and periodontitis. A Korean study recently revealed a correlation between an increased prevalence of chronic periodontitis and worsening of NAFLD scores [20]. Further studies demonstrated that there might be an association between NAFLD and elevated rates of periodontitis, tooth loss and untreated caries among adults in the United States [21] and identified periodontitis as a possible risk factor for disease progression to fibrosis in Japanese patients with NAFLD and obesity [33]. On the other way round, a Japanese study showed an improvement in AST and alanine aminotransferase (ALT) levels after three months of non-surgical periodontal treatment compared with baseline levels in 10 individuals with NAFLD and periodontitis [34].

It is proposed that oral bacteria may have an impact on liver tissue. The possible mechanisms underlying the connection between periodontal pathogenic microbes and MASLD are described under the term “oral–gut–liver axis”, which has been discussed in multiple reviews [23, 35, 36]. It is hypothesised that oral bacteria can be translocated to the gut causing intestinal dysbiosis [23]. Consequently, these changes in microbiota might lead to increased amounts of hepatotoxic molecules in the gut and impair the intestinal barrier which increases its permeability [23]. Bacterial components can thus be translocated to the liver, where they may cause damage to hepatic tissue [35]. The composition of the oral microbiome therefore seems to influence the pathogenesis of MASLD through intestinal mucosa linking it to the liver.

The connection between oral hygiene and the oral microbiome was examined by an Irish study [37]. They reported that tooth brushing at least once a day was related to a greater diversity of mucosal species while poor oral hygiene, measured by the simplified oral hygiene index (OHI-S), was correlated with an increased number of periodontitis-associated bacteria [37]. These findings illustrate that oral hygiene practices, such as tooth brushing, can modulate the oral microbiome, which may provide an explanation for their potential influence on the pathogenesis of MASLD.

Good oral hygiene behaviours with a tooth brushing frequency of at least twice a day were previously shown to have beneficial effects on the drive of cardiometabolic diseases such as type 2 diabetes mellitus, cardiovascular disease and chronic kidney disease as summarised in a systematic review in 2024 [38]. This supports the assumption that there could also be a link to MASLD. To our knowledge this is the first systematic review assessing the association between oral hygiene measures and the course of MASLD/MASH.

There are some limits to that review, however. Only a few studies about this topic have been published so far, and they all presented deficiencies in study quality. A weak point was that self-reports to measure the exposure (tooth brushing frequency) were used, which can introduce bias. Moreover, tooth brushing frequency alone is a limited indicator on oral hygiene, as other factors, such as brushing technique, the type of toothbrush used or interdental cleaning, must be considered as well. Rather than evaluating oral hygiene by participant self-report, future studies should employ more objective and comparable methods, such as standardised oral hygiene indices assessed by dental professionals. In addition to tooth brushing, Gheisary et al. investigated other oral hygiene behaviours, namely the use of dental floss and mouthwash, but also based on self-report [28]. Pischke et al. did not report a way to address confounding factors that could have an influence on the outcome [27]. The only cohort study excluded all participants without a second health check-up during the follow-up period and therefore excluded them from data analysis [31]. Excluding people with no follow-up reduces missing outcome data for the included cohort, but risks selection bias because those excluded might differ systematically. Regarding comparability of the studies, it is to mention that they used different methods to diagnose NAFLD/NASH. As all the included studies were of observational nature, most of them cross-sectional and retrospective, while no randomised-controlled trial has been conducted, establishing causality is not possible. Furthermore, the loss of statistical significance after adjustment for confounding variables in two of the included studies suggests that factors other than oral hygiene, such as socio-demographic parameters or metabolic conditions, may have contributed substantially to the observed associations with liver disease. Gheisary et al. excluded individuals with cardiometabolic diseases like diabetes mellitus [28]. However, significant differences in body mass index (BMI) and education levels were observed between the NAFLD and control groups. Participants with NAFLD had a higher mean BMI and lower education levels than controls, which are both factors that could have a major influence on the risk of developing NAFLD. The study did not clearly report whether these variables were considered or adjusted for in the analysis.

Conducting interventional studies on this topic may present ethical difficulties, particularly when study designs involve restricting recommended oral hygiene practices. Nonetheless, more research, e.g. in the form of prospective cohort studies, is needed. Such studies should employ more standardised and objective methods of oral health assessment and address the influence of potential covariates such as socio-demographic parameters or health-related habits and conditions.

Among the six included studies, different diagnostic tools were used to detect the presence or progression of NAFLD/NASH. Kim et al. used the FLI to diagnose NAFLD in participants [30]. This non-invasive index was developed to assess hepatic steatosis and is easily available as it is calculated by BMI, waist circumference, triglyceride and gamma-glutamyltransferase (GGT) levels [39]. Its application is limited, however, as it does not allow differentiation between the stages of steatosis and does not have the same accuracy as liver biopsy or imaging tools [39]. Yamamoto et al. and Gheisary et al. used ultrasonography to diagnose NAFLD, which is one of the methods recommended by the European Association for the Study of the Liver (EASL) [9, 28, 31]. However, when it comes to steatohepatitis, liver biopsy remains the gold standard as it is the only way to evaluate histologic features [9]. On the other hand, it is an invasive method and therefore not suitable for routine examination [9]. To assess disease progression more easily available, non-invasive tests like Fibrosis-4 (FIB-4) or NAFLD fibrosis score (NFS) as well as imaging techniques can be helpful and are recommended by the EASL [9]. The FIB-4 score and NFS have been validated for detecting fibrosis and can be combined with imaging methods such as LSM based on transient elastography [9, 39]. Pischke et al. and Keklikkiran et al. both used transient elastography for LSM in their studies to detect fibrosis progression in NASH patients [27, 29]. Ram et al. did not state the diagnostic tool used to detect NAFLD prevalence [32].

The current evidence is insufficient to establish a causal relationship between oral hygiene habits and MASLD. Nevertheless, despite the limitations of the available studies, this review emphasises the significance of good oral hygiene and its potentially beneficial association with liver health. A recent study from the UK found out that bad oral health conditions were associated with a higher incidence of MASLD in adjusted models [40]. Regardless of their genetic predisposition, there was a significantly increased risk to develop MASLD among participants with poor oral health [40]. Against this background, frequent tooth brushing and regular dental check-ups as the foundation of oral health maintenance are becoming increasingly important.

## Conclusions

Current evidence indicates that oral health is strongly related to overall health. Indeed, this systematic review pointed out that frequent oral hygiene measures were associated with a lower risk, prevalence or progression of NAFLD or NASH. Although the current preliminary evidence does not yet allow to establish a causal connection between MASLD and oral hygiene practices, these findings support the potential importance of good oral hygiene and regular dental visits, particularly for patients with fatty liver disease. To clearly understand the nature and underlying mechanisms of this association as well as the influence of potential cofactors more research is needed.

## Supplementary information

Supplementary Material 1.

Supplementary Material 2.

Supplementary Material 3.

## Data Availability

All data generated or analysed during this study are included in this published article and its supplementary information files.

## Abbreviations

ALD: Alcohol-associated Liver Disease
ALT: Alanine Aminotransferase
AST: Aspartate Aminotransferase
BMI: Body Mass Index
EASL: European Association for the Study of the Liver
FIB-4: Fibrosis-4
FLI: Fatty Liver Index
GGT: Gamma-glutamyltransferase
JBI: Joanna Briggs Institute
LSM: Liver Stiffness Measurement
MASH: Metabolic Dysfunction-associated Steatohepatitis
MASLD: Metabolic Dysfunction-associated Steatotic Liver Disease
MELD: Model for End-stage Liver Disease
NAFLD: Non-alcoholic Fatty Liver Disease
NASH: Non-alcoholic Steatohepatitis
NFS: NAFLD Fibrosis Score
OHI-S: Simplified Oral Hygiene Index
PRISMA: Preferred Reporting Items for Systematic Reviews and Meta-Analyses
SLD: Steatotic Liver Diseases
