# Supplementary material for: The association between oral hygiene and metabolic dysfunction-associated steatotic liver disease – a systematic review
Source: BMC Oral Health. 2026 Jul 8;26:1238. doi: 10.1186/s12903-026-09067-y (PMC13353020; doi:10.1186/s12903-026-09067-y)
Supplement: Supplementary file 1 — Supplementary Material 1. [file 12903_2026_9067_MOESM1_ESM.docx]

**Supplemental File 1**

Supplementary material containing detailed information on the search strategy, search term and additional information on data extraction.

**PICO criteria and search strategy**

The PICO criteria for study selection were defined as follows:

- **Participants:** Regarding participants, we included studies investigating adults (18 years or older) as well as children if data for this age group were reported separately. Study participants could be composed either of individuals with MASLD/NAFLD, MASH/NASH, non-exposed subjects or a combination of all. When examining liver diseases in general only studies distinguishing between MASLD/NAFLD and other causes of chronic liver disease were included. Studies that focused on patients with liver cirrhosis or end stage liver disease with unknown aetiology were excluded.
- **Interventions:** Concerning interventions, eligible studies assessed the impact of oral hygiene measures such as tooth brushing frequency or frequency of dental visits. This information could be based on self-reports by participants.
- **Comparison:** We included studies that compared participants with a higher frequency of oral hygiene practices with those with a lower frequency. Control groups were defined as having a lower daily tooth brushing frequency, fewer annual dental visits or lower frequency of any other oral hygiene measures.
- **Outcome:** Analysed outcomes were the presence of MASLD as well as changes or differences in liver health including steatosis, fibrosis and inflammation, as measured by non-invasive or invasive methods or by biomarkers for liver function that either indicate a disease improvement or progression in follow-up period. In individuals who were not exposed at baseline, the evaluated outcome was whether the previously described features indicate the incidence of MASLD during follow-up. No restrictions were imposed on the follow-up periods.

**Search term**

| **Search** | **Query** |
| --- | --- |
| *#1* | (oral hygiene OR tooth brushing OR dental visit OR periodont*) |
| *#2* | (fatty liver OR nash OR mash OR nafld OR masld) |
| *#3* | #1 AND #2 |

*WEB OF SCIENCE*

TS=((oral hygiene OR tooth brushing OR dental visit OR periodontal) AND (fatty liver OR nash OR mash OR nafld OR masld))

🡪 Filter: Database: all but MEDLINE, Languages: English, Document Types: Article

*SCOPUS*

TITLE-ABS-KEY (( oral hygiene OR tooth brushing OR dental visit OR periodont* ) AND ( fatty liver OR nash OR mash OR nafld OR masld ))

*EMBASE*

((oral hygiene or tooth brushing or dental visit or periodontal) and (fatty liver or mash or nash or nafld or masld)).mp. [mp=title, abstract, heading word, drug trade name, original title, device manufacturer, drug manufacturer, device trade name, keyword heading word, floating subheading word, candidate term word]

🡪 Filter: Medline removed

*PUBMED*

((oral hygiene) OR (tooth brushing) OR (dental visit) OR (periodontal)) AND ((fatty liver) OR (mash) OR (nash))

*("oral hygiene"[MeSH Terms] OR ("oral"[All Fields] AND "hygiene"[All Fields]) OR "oral hygiene"[All Fields] OR ("toothbrushing"[MeSH Terms] OR "toothbrushing"[All Fields] OR ("tooth"[All Fields] AND "brushing"[All Fields]) OR "tooth brushing"[All Fields]) OR (("dental health services"[MeSH Terms] OR ("dental"[All Fields] AND "health"[All Fields] AND "services"[All Fields]) OR "dental health services"[All Fields] OR "dental"[All Fields] OR "dentally"[All Fields] OR "dentals"[All Fields]) AND ("visit"[All Fields] OR "visitation"[All Fields] OR "visitations"[All Fields] OR "visited"[All Fields] OR "visiting"[All Fields] OR "visits"[All Fields])) OR ("periodontal"[All Fields] OR "periodontally"[All Fields] OR "periodontically"[All Fields] OR "periodontics"[MeSH Terms] OR "periodontics"[All Fields] OR "periodontic"[All Fields] OR "periodontitis"[MeSH Terms] OR "periodontitis"[All Fields] OR "periodontitides"[All Fields])) AND ("fatty liver"[MeSH Terms] OR ("fatty"[All Fields] AND "liver"[All Fields]) OR "fatty liver"[All Fields] OR "mash"[All Fields] OR ("non alcoholic fatty liver disease"[MeSH Terms] OR ("non alcoholic"[All Fields] AND "fatty"[All Fields] AND "liver"[All Fields] AND "disease"[All Fields]) OR "non alcoholic fatty liver disease"[All Fields] OR "nash"[All Fields]))*

**Data extraction**

The extracted information of each study, when present, comprised the following:

- Name of first author
- Year and country of publication
- Study aim
- Study design and methodology including sample number, participant demographics and baseline characteristics
- Type of exposure
- Methods of assessment of MASLD and oral hygiene
- Study outcomes
- Study findings/conclusion
