# Supplementary material for: The association between oral hygiene and metabolic dysfunction-associated steatotic liver disease – a systematic review
Source: BMC Oral Health. 2026 Jul 8;26:1238. doi: 10.1186/s12903-026-09067-y (PMC13353020; doi:10.1186/s12903-026-09067-y)
Supplement: Supplementary file 2 — Supplementary Material 2. [file 12903_2026_9067_MOESM2_ESM.docx]

**Supplemental File 2**

**Risk of Bias Assessment of Included Studies**

| **JBI Critical Appraisal Checklist for Analytical Cross-sectional Studies** |  |  |  |  |  |
| --- | --- | --- | --- | --- | --- |
| STUDY | Kim et al., 2021 | Ram et al., 2022 | Keklikkiran et al., 2023 | Pischke et al., 2023 | Gheisary et al., 2026 |
| **1. Were the criteria for inclusion in the sample clearly defined?** |  |  |  |  |  |
| a) yes | x | x | x | x | x |
| b) no |  |  |  |  |  |
| c) unclear |  |  |  |  |  |
| d) not applicable |  |  |  |  |  |
| **2. Were the study subjects and the setting described in detail?** |  |  |  |  |  |
| a) yes | x | x | x | x | x |
| b) no |  |  |  |  |  |
| c) unclear |  |  |  |  |  |
| d) not applicable |  |  |  |  |  |
| **3. Was the exposure measured in a valid and reliable way?** |  |  |  |  |  |
| a) yes |  |  |  |  |  |
| b) no | x | x | x | x | x |
| c) unclear |  |  |  |  |  |
| d) not applicable |  |  |  |  |  |
| **4. Were objective, standard criteria used for measurement of the condition?** |  |  |  |  |  |
| a) yes | x |  | x | x | x |
| b) no |  |  |  |  |  |
| c) unclear |  | x |  |  |  |
| d) not applicable |  |  |  |  |  |
| **5. Were confounding factors identified?** |  |  |  |  |  |
| a) yes | x | x | x | x | x |
| b) no |  |  |  |  |  |
| c) unclear |  |  |  |  |  |
| d) not applicable |  |  |  |  |  |
| **6. Were strategies to deal with confounding factors stated?** |  |  |  |  |  |
| a) yes | x | x | x |  |  |
| b) no |  |  |  | x |  |
| c) unclear |  |  |  |  | x |
| d) not applicable |  |  |  |  |  |
| **7. Were the outcomes measured in a valid and reliable way?** |  |  |  |  |  |
| a) yes | x |  | x | x | x |
| b) no |  |  |  |  |  |
| c) unclear |  | x |  |  |  |
| d) not applicable |  |  |  |  |  |
| **8. Was appropriate statistical analysis used?** |  |  |  |  |  |
| a) yes | x | x | x | x | x |
| b) no |  |  |  |  |  |
| c) unclear |  |  |  |  |  |
| d) not applicable |  |  |  |  |  |
| Overall yes | 7 | 5 | 7 | 6 | 6 |

| **JBI Critical Appraisal Checklist for cohort studies** |  |
| --- | --- |
| STUDY | Yamamoto et al., 2021 |
| **1. Were the two groups similar and recruited from the same population?** |  |
| a) yes | x |
| b) no |  |
| c) unclear |  |
| d) not applicable |  |
| **2. Were the exposures measured similarly to assign people to both exposed and unexposed groups?** |  |
| a) yes | x |
| b) no |  |
| c) unclear |  |
| d) not applicable |  |
| **3. Was the exposure measured in a valid and reliable way?** |  |
| a) yes |  |
| b) no | x |
| c) unclear |  |
| d) not applicable |  |
| **4. Were confounding factors identified?** |  |
| a) yes | x |
| b) no |  |
| c) unclear |  |
| d) not applicable |  |
| **5. Were strategies to deal with confounding factors stated?** |  |
| a) yes | x |
| b) no |  |
| c) unclear |  |
| d) not applicable |  |
| **6. Were the groups/participants free of the outcome at the start of the study (or at the moment of exposure)?** |  |
| a) yes | x |
| b) no |  |
| c) unclear |  |
| d) not applicable |  |
| **7. Were the outcomes measured in a valid and reliable way?** |  |
| a) yes | x |
| b) no |  |
| c) unclear |  |
| d) not applicable |  |
| **8. Was the follow up time reported and sufficient to be long enough for outcomes to occur?** |  |
| a) yes |  |
| b) no |  |
| c) unclear |  |
| d) not applicable | x |
| **9. Was follow up complete, and if not, were the reasons to loss to follow up described and explored?** |  |
| a) yes |  |
| b) no |  |
| c) unclear | x |
| d) not applicable |  |
| **10. Were strategies to address incomplete follow up utilized?** |  |
| a) yes |  |
| b) no |  |
| c) unclear | x |
| d) not applicable |  |
| **11. Was appropriate statistical analysis used?** |  |
| a) yes | x |
| b) no |  |
| c) unclear |  |
| d) not applicable |  |
| Overall yes | 7 |
