# Supplementary material for: The association between oral hygiene and metabolic dysfunction-associated steatotic liver disease – a systematic review
Source: BMC Oral Health. 2026 Jul 8;26:1238. doi: 10.1186/s12903-026-09067-y (PMC13353020; doi:10.1186/s12903-026-09067-y)
Supplement: Supplementary file 3 — Supplementary Material 3. [file 12903_2026_9067_MOESM3_ESM.docx]

**Supplemental File 3**

**Detailed data extraction of included studies**

| First author, year | Main findings |
| --- | --- |
| Kim, 2021 | - decreasing FLI with increasing tooth brushing (tb) frequency 🡪 mean FLI in relation to tb frequency   (p < 0.001):   - - tb less than 1x/d: FLI 20.3   - tb 2x/d: FLI 15.7   - tb more than 3x/d: FLI 11.0 - significant negative correlation of FLI ≥ 60 with higher tb frequency (adjusted odds ratio (OR)) 🡪 multiple logistic regression analysis, adjustment for:   - age, sex (model 1): OR for FLI ≥ 60 (compared to tb ≤ 1x/d (p = 0.02)) tb 2x/d: 0.65; tb ≥ 3x/d: 0.52   - + smoking, regular exercise, current alcohol consumption, income, education level (model 2): OR for FLI ≥ 60 (compared to tb ≤ 1x/d (p = 0.034)) tb 2x/d: 0.66; tb ≥ 3x/d: 0.54   - no significant correlation when additionally adjusting for diabetes mellitus or regular dental check-ups (p > 0.05) - no significant correlation between regular dental check-ups and OR for FLI ≥ 60 compared to no regular dental check-ups (p > 0.05) |
| Yamamoto, 2021 | NAFLD risk decreases with increasing tb frequency 🡪 multivariate longitudinal analysis (adjusted for age, sex, BMI, smoking, exercise, underlying health conditions): OR for developing NAFLD in relation to tb frequency (compared to tb ≤ 1x/d (p < 0.05)): tb 1-2x/d: 0.85; tb 3x/d: 0.74 |
| Ram, 2022 | - significant relation of higher tb frequency (≥ 1x/d) with lower NAFLD prevalence 🡪 binary logistic regression: OR for tb ≥ 1x/d among NAFLD patients (compared to individuals without NAFLD (p < 0.001)): 0.50 - loss of statistical significance between NAFLD and tb frequency (p > 0.05) when controlling for socio-demographic parameters, health-related risk habits (e.g. smoking), conditions associated with the metabolic syndrome, and auxiliary (blood) test results in multivariate logistic regression analysis |
| Pischke, 2023 | significantly increased liver parameters (AST, MELD score and liver stiffness values) among NASH patients without regular dental visits (≤ 1x/y) compared to those with regular check-ups (> 1x/y) |
| Keklikkiran, 2023 | tb frequency ≤ 1/d associated with higher risk for LSM ≥ 12kPa compared to tb 2x/d or after every meal 🡪 multivariable logistic regression: OR 1.69  (p = 0.0236) |
| GHEISARY, 2026 | lower frequency of tb, flossing and using mouth wash in the NAFLD group compared with the control group:   - tb: 18.6% never in the NAFLD group, 0% never in the control group (p = 0.006); 11.6% 2x/d in the NAFLD group, 51.2% in the control group (p < 0.001) - flossing: 58.1% never in the NAFLD, 0% never in the control (p < 0.001) - mouthwash: never 72.1% in the NAFLD group, 0% in the control group (p < 0.001) |
